# International consensus (ICON) on: clinical consequences of mite hypersensitivity, a global problem

**DOI:** 10.1186/s40413-017-0145-4

**Published:** 2017-04-18

**Authors:** Mario Sánchez-Borges, Enrique Fernandez-Caldas, Wayne R. Thomas, Martin D. Chapman, Bee Wah Lee, Luis Caraballo, Nathalie Acevedo, Fook Tim Chew, Ignacio J. Ansotegui, Leili Behrooz, Wanda Phipatanakul, Roy Gerth van Wijk, Demoly Pascal, Nelson Rosario, Motohiro Ebisawa, Mario Geller, Santiago Quirce, Susanne Vrtala, Rudolf Valenta, Markus Ollert, Giorgio Walter Canonica, Moises A. Calderón, Charles S. Barnes, Adnan Custovic, Suwat Benjaponpitak, Arnaldo Capriles-Hulett

**Affiliations:** 1 Allergy and Clinical Immunology Department, Centro Médico Docente La Trinidad, Caracas, Venezuela; 2 Clínica El Avila, 6ª transversal Urb. Altamira, Piso 8, Consultoria 803, Caracas, 1060 Venezuela; 3 Inmunotek S.L., Madrid, Spain and Division of Allergy and Immunology, University of South Florida College of Medicine, Tampa, FL USA; 4 Telethon Kids Institute, University of Western Australia, Crawley, WA Australia; 5 Indoor Biotechnologies, Charlottesville, VA USA; 6 Department of Paediatrics, Yong Loo Lin School of Medicine, National University of Singapore, Singapore, Singapore; 7 Institute for Immunological Research, University of Cartagena, Cartagena, Colombia; 8 University of Cartagena, Cartagena, Colombia; 9 Department of Biological Sciences, Allergy and Molecular Immunology Laboratory, Functional Genomics Laboratories, National University of Singapore, Singapore, Singapore; 10 Department of Allergy and Immunology, Hospital Quirón Bizkaia, Bilbao, Spain; 11 Division of Immunology and Allergy, Boston Cshildren’s Hospital, Harvard Medical School, Boston, MA USA; 12 Department of Internal Medicine, Allergology, Erasmus MC, Rotterdam, the Netherlands; 13 Division of Allergy, Department of Pulmonology, University Hospital of Montpellier, Paris, France; 14 Montpellier and Pierre Louis Institute of Epidemiology and Public Health, Sorbonne Universités, Paris, France; 15 Federal University of Parana, Rua General Carneiro, Curitiba, Brazil; 16 Department of Allergy, Clinical Research Center for Allergology and Rheumatology, Sagamihara National Hospital, Sagamihara, Kanagawa Japan; 17 Division of Medicine, Academy of Medicine of Rio de Janeiro, Rio de Janeiro, Brazil; 18 Department of Allergy, Hospital La Paz Institute for Health Research and CIBER of Respiratory Diseases (CIBERES), Madrid, Spain; 19 Division of Immunopathology, Department of Pathophysiology and Allergy Research, Center for Pathophysiology, Infectiology and Immunology, Medical University of Vienna, Vienna, Austria; 20 Department of Infection & Immunity, Laboratory of Immunogenetics and Allergology, Luxembourg Institute of Health, Luxembourg, UK; 21 Allergy & Respiratory Diseases Clinic, University of Genoa, IRCCS AOU San Martino-IST, Genoa, Italy; 22 Section of Allergy and Clinical Immunology, Imperial College London – NHLI, London, United Kingdom; 23 Division of Allergy/Immunology, Children’s Mercy Hospital, Kansas City, MO USA; 24 Department of Paediatrics, Imperial College London, London, United Kingdom; 25 Division of Pediatric Allergy/Immunology/Rheumatology, Department of Pediatrics, Ramathibodi Hospital, Mahidol University, Bangkok, Thailand

## Abstract

Since mite allergens are the most relevant inducers of allergic diseases worldwide, resulting in significant morbidity and increased burden on health services, the International Collaboration in Asthma, Allergy and Immunology (iCAALL), formed by the American Academy of Allergy, Asthma and Immunology (AAAAI), the American College of Allergy, Asthma and Immunology (ACAAI), the European Academy of Allergy and Clinical Immunology (EAACI), and the World Allergy Organization (WAO), has proposed to issue an International Consensus (ICON) on the clinical consequences of mite hypersensitivity. The objectives of this document are to highlight aspects of mite biology that are clinically relevant, to update the current knowledge on mite allergens, routes of sensitization, the genetics of IgE responses to mites, the epidemiologic aspects of mite hypersensitivity, the clinical pictures induced by mites, the diagnosis, specific immunotherapeutic approaches, and prevention.

## Introduction

Mite allergens are able to sensitize and induce allergic symptoms in sensitized and genetically predisposed individuals resulting in allergic rhinoconjunctivitis, asthma, and atopic dermatitis. . The main sources of allergens in house dust worldwide are the fecal pellets of the mite species *Dermatophagoides pteronyssinus, Dermatophagoides farinae, Euroglyphus maynei,* and the storage mites *Blomia tropicalis, Lepidoglyphus destructor* and *Tyrophagus putrescentiae*. Recent advances in the study of mite biology, mite allergen properties and cross-reactivities, have provided better approaches for the prevention and management of diseases produced by exposure to mite allergens, including improved diagnostic and immunotherapeutic methods [1]. In this ICON document a comprehensive review of the major issues concerning human diseases caused by mites will be presented, as a contribution to the understanding of the importance of mite hypersensitivity as an important inducer of diseases that constitute a major public health concern all around the world.

## Methodology

A working committee that included members of the participating organizations who are actively working in the field of mite hypersensitivity was formed, taking into account regional representation and previous individual publications. Members were assigned sections which, when completed, were compiled by the project leader and circulated to all members. After corrections were included the final draft was submitted for approval by the governing boards of the participating organizations.

## Overview of biology

**Fig. 1:**
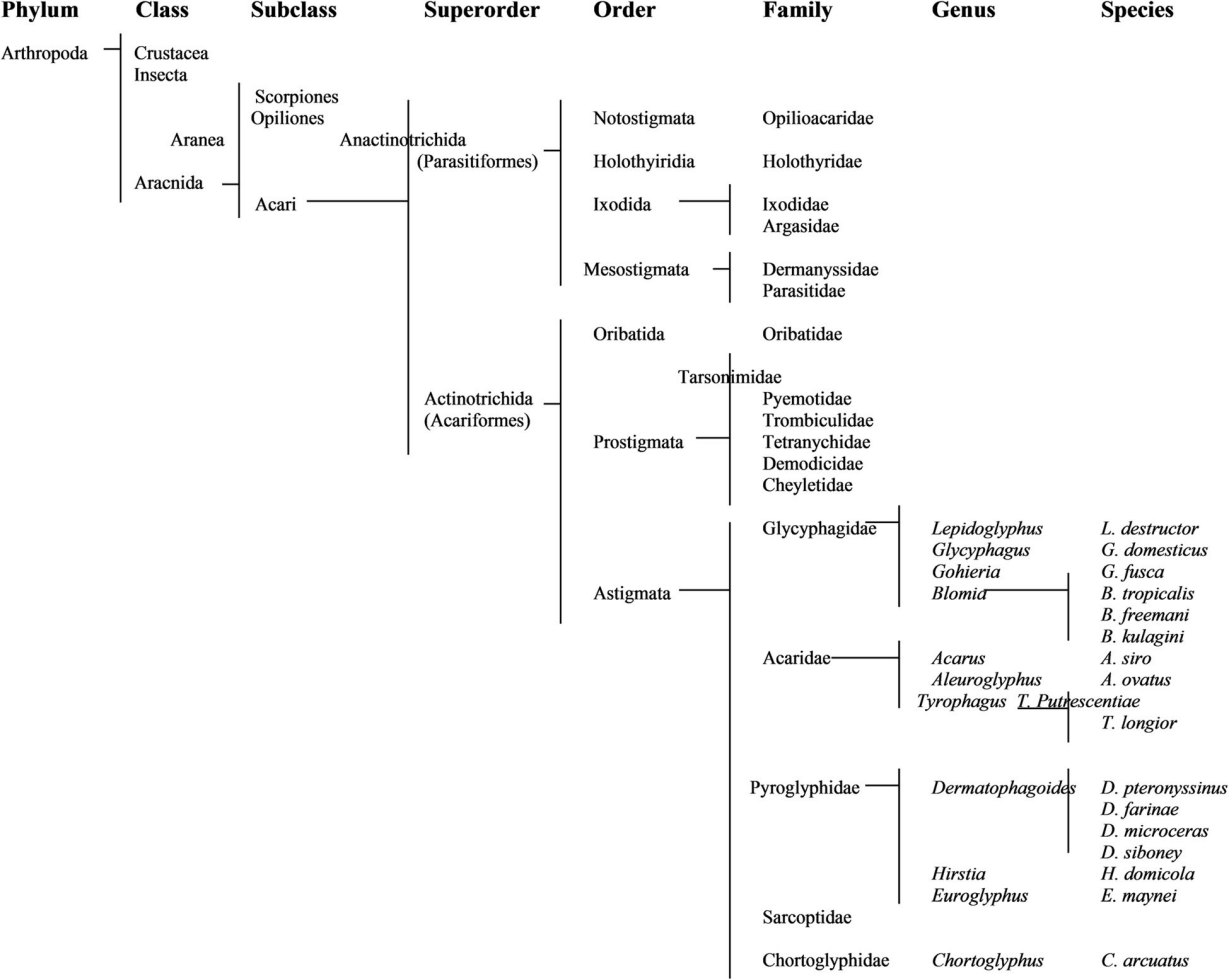
Taxonomy of Mites with Allergenic Relevance

Mites and ticks are included in the subclass Acari, which forms one of the most abundant and diverse biological groups within the arachnids (Arthropods) (Fig. 1). Its name derives from the Latin word acarus, which in turn derives from the Greek name Akari, whose first known mention is attributed to Aristotle (, Historia Animalium, Book 5, Chapter 32). He most likely used this name to mention the mite species *Carpoglyphis lactis*. Mites are characterized by having four pairs of legs, such as spiders and scorpions (insects have three pairs of legs; crustaceans five pairs of legs) which are relatively smaller than their bodies. Most of the mite species have a size of less than 1 mm (most house dust mite species are below half a millimeter). Mites can be considered as one of the oldest terrestrial animals. One of the first known mite fossils (identified as *Protoacarus crani,* Raford) originates from the Devonian period, nearly 400 Ma ago. Approximately 30,000 species of mites, placed in more than 1,700 genera, have been described. However, it is considered that the number of undescribed species could be more than 100,000. Mites are distributed worldwide and have competed with insects for aquatic and terrestrial habitats. They can be found in the soil of forests and grasslands, or on any organic waste. Many species spend part of their life cycle in trees and shrubs, while others live in caves or have adapted to live in hot springs. Depending on the species, they feed on plants, fungi, algae, organic matter, animal waste, other arthropod, nematode, or infest the exterior and interior of all kinds of animals (insects, reptiles, birds and mammals) [2].

Most domestic mites belong to the order Astigmata. They do not have external respiratory openings or stigmata (Astigmata means “no stigma”). Their small size makes the ratio between the body surface and its interior sufficient to allow efficient gas exchange through the skin. In other orders, breathing could also take place through body openings (1–4 stigmas), which are located in the front half of the body, or through a trachea [3].

Mites have developed a digestive tract, including a mouth and mouth parts, salivary glands, and an intestine, consisting of esophagus, small intestine, along with a large intestine and an anal opening. Their digestive system produces spherical fecal particles (diameter of about 20 μm) wrapped in a peritrophic membrane, which are the main vector of mite allergens. Mites most commonly found inside the houses worldwide include *D. pteronyssinus, D. farinae, E. maynei and B. tropicalis* [4, 5]. They feed mainly on human scales and other micronutrients. The amount of human flakes released daily by an adult is of 0.5 to 1 g, and therefore, it is a very abundant food source. In addition to human skin scales the diet may also include: fungi and other constituents of the skin microbiota, body fragments of insects (beetles, cockroaches, moths), etc.

The life cycle of dust mites and some of the storage mites consists of five stages (egg, larva, protonymph, tritonymph and adults). In each stage, there is an active period followed by another, shorter, quiescent stage, before a new stadium emerges from the old exoskeleton. The quiescent period protonymph in dust mites can be long lasting as it is resistant to drying and allows mites to survive long dry periods (several months). This stage remains attached to the substrate and cannot be removed by vacuuming. Some storage mites (*L. destructor, A. siro*) have an additional stage (deutonymph or hipopus) which allows them to resist unfavorable weather or nutritional conditions.

The life cycle of the mites is directly dependent on the temperature. Microhabitats where mites are found in homes are not uniform in temperature and relative humidity and the temperature fluctuates within a microhabitat. Thus, their development at low-temperature (on the floor) is lower as compared to their development in warmer conditions (mattresses, or sofas). For *D. pteronyssinus*, a cycle from egg to adult takes about 122 days at 16 °C (75% RH) while it only takes 15 days at 35 °C [6, 7].

An allergenic role has been attributed to house dust since the early years of the past century. This allergenicity was responsible for a large number of respiratory allergic diseases worldwide. The importance of these allergic manifestations induced by the inhalation of house dust present in soil, mattresses, carpets, rugs, sofas, and comforters was thoroughly studied by different researchers in an attempt to identify the main components responsible for this allergenic effect. In 1921, Kern refers for the first time to the importance of house dust in allergic manifestations [8]. In 1922, Cooke speculates about the existence of allergens of unknown origin and nature in house dust allergen and extracts were prepared for desensitization studies [9]. In 1924, Storm Van Leeuwen associated the dust allergy phenomenon with certain climatic circumstances, as spectacular clinical improvements were observed when patients were moved to high mountain climates with low relative humidities [10]. The occasional discovery of mites in house dust was pointed out by different investigators on several occasions. The presence in house dust of mites of the genus *Dermatophagoides farinae* was indicated for the first time in 1964 by Oshima [11]. However, Voorhorst and Spieksma in 1964, showed that house dust contains mite species with a high allergenic power, which could be responsible for the allergenicity of house dust [12]. Fain identified in 1966 the mite *D. pteronyssinus* as the main allergen source responsible for numerous respiratory allergies induced by the inhalation of house dust [13].

Mites found worldwide in human premises can generally be grouped into house dust mites and storage mites. They are found in carpets, fabrics, upholstery, pillows and mattresses. *Blomia tropicalis,* was formerly known as a storage dust mite, but is now also accepted as a house dust mite as it is found extensively in dust from homes in tropical and subtropical countries. Storage mites include *Glycyphagus domesticus, Lepidoglyphus destructor, Blomia kulagini, Tyrophagus putrescentiae, Acarus siro, Suidasia pontifica, Glycycometus malaysiensis, Aleuroglyphus ovatus* and *Thyreophagus entomophagus*. Storage mites are commonly found worldwide in storage facilities for grains such as wheat, corn, oats, barley and hay. They may contaminate or invade and thrive in processed foods made from the grains (e.g., flour, cereals and baking mixes) when these products become moist or are stored in humid environments. In recent years, the new term domestic mites has been coined to include all mite species present in the indoor environment that can sensitize humans. It includes all the above mentioned species which can be regularly found in the indoor environment, including bedding, sofas, kitchen floors, etc.

Mite identification studies worldwide have confirmed that most mite species are present in most sites where these studies have been conducted, including the Northern and Southern hemispheres. Mites are almost absent in the artic regions, or in highly cold and dry climates, such as in high altitude areas in the Alps. There is ample evidence that many mite species can sensitize exposed individuals and produce allergic diseases. It has been suggested that any mite species which is in contact with a genetically prone individual, can induce sensitization. Mite allergens can be detected in many areas of the home, including beds, carpets, upholstered furniture and clothing. Leather-covered couches, wood furniture, and bare floors contain fewer mites. Beds are the perfect habitat for mites, since they provide the ideal temperature, food and moisture for their proliferation, and allergens they produce accumulate deep inside mattresses and pillows, especially when they are old. Information on the distribution of house dust mites provides valuable data to design environmental control strategies.

Allergy to mites is a global health problem recognized by the World Health Organization, which affects millions of people around the world. The discovery of the cause-effect relationship between sensitization to mites and asthma is relatively recent, approximately 50 years. In these years there have been significant advances in the identification and characterization of mite allergens and many have been purified and sequenced. Similarly, there has been great progress in the standardization of allergenic extracts of several mite species for diagnosis and treatment, and the clinical efficacy of immunotherapy using extracts of several species of mites has been demonstrated.

## Mite allergens

**Fig. 2:**
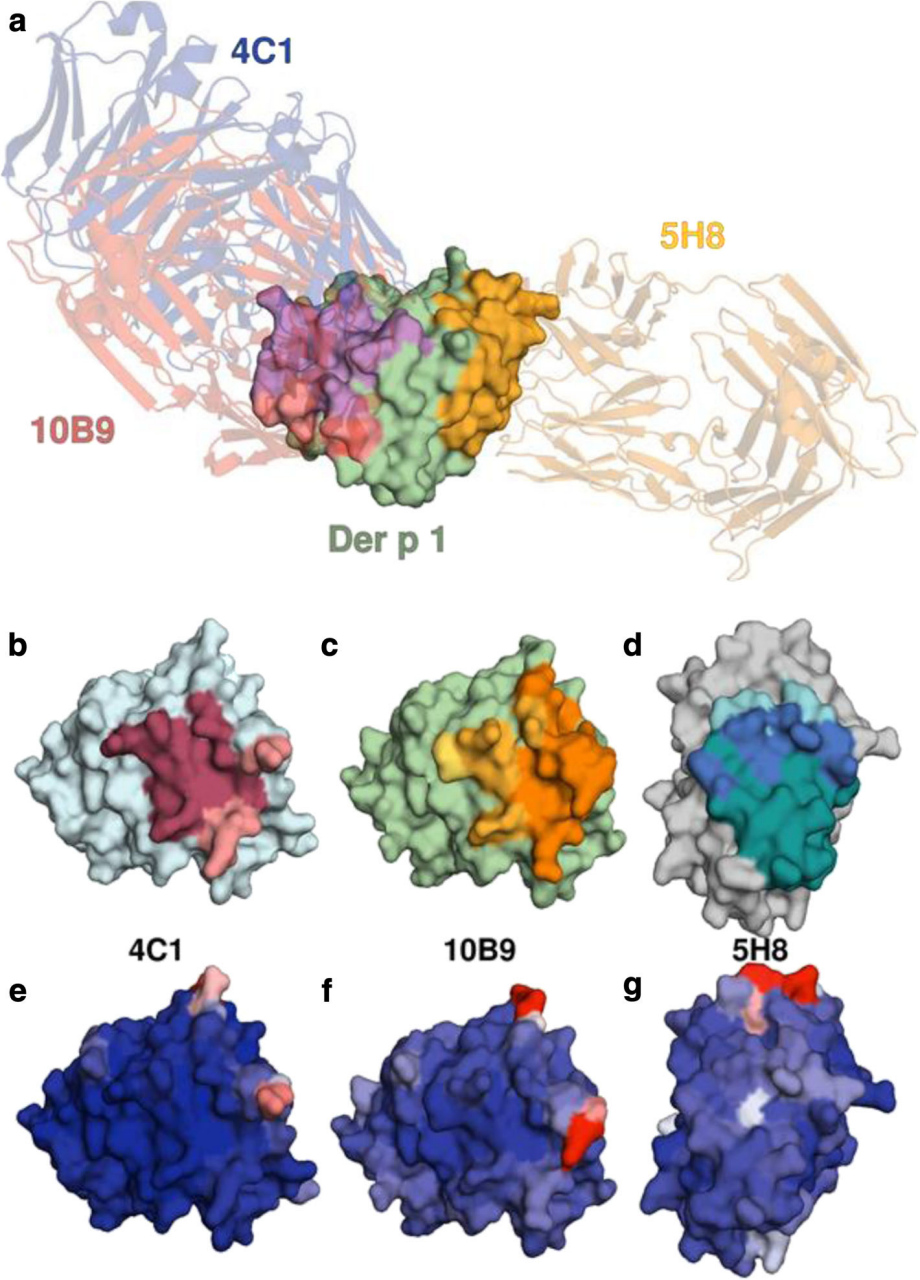
X-ray crystal structure of Der p 1 in complex with Fab fragments of three anti-Der p 1 monoclonal antibodies, 5H8, 10B9, and 4C1. Reproduced from: Tomasz Osinski, Anna Pomés, Karolina A. Majorek, Jill Glesner, Lesa R. Offermann, Lisa D. Vailes, Martin D. Chapman, Wladek Minor, Maksymilian Chruszcz. Structural analysis of Der p 1-antibody complexes and comparison with complexes of proteins or peptides with monoclonal antibodies. *J Immunol* 2015; 195: 307–316. DOI: 10.4049/jimmunol.1402199. Figure 1 reused with permission. Copyright 2015. The American Association of Immunologists, Inc

The World Health Organization and the International Union of Immunological Societies (WHO/IUIS) allergen nomenclature sub-committee currently includes up to 31 *D. pteronyssinus* and *D. farinae allergens* in the systematic nomenclature, as well as 13 allergens from *Blomia tropicalis* and multiple allergens from storage mite species [14]. Most of these allergens have been defined by IgE binding or skin test reactivity and have been cloned, sequenced and expressed as recombinant proteins for further analyses [15]. The three dimensional structures of several important allergens have been determined by X-ray crystallography or nuclear magnetic resonance spectroscopy and the atomic coordinates have been deposited in the protein database (PDB) (Fig. 2) [16].

The finding that the individual allergen components make characteristic contributions to the overall anti-mite IgE response of most allergic subjects [15] suggests that the biological functions of the individual mite allergens and how they are delivered contribute to their allergenicity. The group 1 allergens of Dermatophagoides spp. (Der p 1 and Der f 1) are cysteine proteases that have, when enzymatically active, the ability to be adjuvants for Th2 inflammatory responses via numerous proposed mechanisms [15, 17]. The apparent low allergenicity of cysteine protease allergens of other mite species, and the susceptibility of cysteine proteases to oxidative deactivation however needs to be considered. The group 2 allergens have structural and functional homology to MD-2, the LPS binding component of Toll Like Receptor 4 (TLR-4). In a mouse model of transient sensitization, Der p 2 plus endotoxin could drive airway Th2 inflammation via TLR-4 and thus could similarly promote IgE antibody responses [18]. In contrast, sensitization via the skin was TLR4 independent in another mouse model [19].

From their structures, other mite allergens (Der p 5, Der p 7, Der p 21) appear to be lipid-binding proteins. These allergens could use attached lipid moieties to interact with the innate immune system or otherwise act as adjuvants for IgE responses [20, 21]. Recently, another major allergen, Der p 23, was identified as a peritrophin homologue associated with the chitinous peritrophic membrane on the surface of mite faeces thus providing an association with the known Th2 adjuvant chitin [22].

The Dermatophagoides spp. group 1, group 2 and group 23 allergens, are immunodominant based on the prevalence and magnitude of IgE responses determined by gravimetric estimations or comparative titrations with proteins of known allergenicity [22–26]. Using these criteria, the group 4, 5, 7 and 21 allergens exhibit medium or mid-tier allergenicity [22, 24–27] and the other groups minor or unknown allergenicity [15, 24, 25]. The group 1 and 2 allergens typically bind 50–70% of the amount of IgE that binds to HDM extracts [23, 24, 26, 27] and while Der p 23 has consistently shown similar IgE binding prevalence to the group 1 and 2 [22, 25–27], not all studies have found high titres [26, 27]. The medium or mid-tier allergens bind IgE in 30–50% of mite-allergic patients and appear to account for most of the residual IgE binding of extracts [15, 24]. The Dermatophagoides spp. allergens are listed in Table 1 in the context of their known allergenicity.

**Table 1:** Allergens from Dermatophagoides species

Immunodominant		Mid-tier		Minor		Unknown	
1	Cysteine protease	4	Alpha amylase	3	Trypsin	14	Large lipid transfer protein
2	ML domain protein	5	Unknown coiled coil bundle	6	Chymotrypsin	22	ML domain protein
23	Peritrophin homologue	7	LPS binding protein homologue	8	Glutathione-S-transferase	24	Cytochrome c reductase binding protein
		21	Group 5 homologue	9	Collagen serine protease	25	Triosephosphatase isomerase
				10	Tropomyosin	26	Myosin alkali light chain
				11	Paramyosin	27	Serpin
				13	Fatty acid binding	28	Heat shock protein
				16	Gelsolin	29	Cyclophilin
				17	Unknown EF hand protein	30	Ferritin
						31	Cofilin
						32	Pyrophosphatase
						33	Alpha tubulin

^a^Group 12 and 19 allergens have not been found for Dermatophagoids spp

^b^No quantitative assessments of IgE binding have been reported for the Unknown groups due to the tests used or the nature of the allergen preparations

Children with stable asthma and those with a propensity to be hospitalised for recurrent and persistent asthma have been found to have a similar profile but with a higher although overlapping amount of IgE binding [24]. However, atopic non-asthmatic subjects have been found to bind IgE to fewer allergens than asthmatics, perhaps due to their overall lower IgE titres [25]. The three immunodominant allergens (Der p 1, Der p 2, Der p 23) also show the highest levels of reactivity in other assessments of allergenicity including skin testing, basophil histamine release assays and RAST inhibition assays [22–28].

The availability of purified allergens, together with sensitive, high-throughput immunoassays (ELISA,MARIA and Microchips) for making allergen measurements, has important clinical applications (Table 2). These assays have direct applications for improving allergy diagnostics and the formulation and testing of therapeutics. Moreover, sophisticated analytical methods, such as mass spectrometry, now enable precise determinations of allergen purity and isoform distribution within mite extracts and within purified allergens themselves. Crystallographic studies have determined the contact residues for monoclonal antibody epitopes on the Group 1 allergens, as well as the surface location of Der p 1 and Der f 1 isoforms [28]. This work is currently being extended to identify IgE antibody binding sites. Progress in structural biology will allow the development of new forms of immunotherapy using strategies based on unmodified recombinant allergens, hypoallergens or T cell peptides.

**Table 2:** Clinical applications of purified mite allergens and assays

•Improved allergen standardization and formulation. To develop HDM extracts with consistent amounts of major allergens for diagnosis and therapy. •To develop formulations of purified allergens for molecular diagnostics with useful discrimination and quantitation of IgE antibody levels and to enable the measurement of allergen-specific IgG antibodies as potential prognostic markers for diagnosis. •To provide environmental exposure assessments to improve patient education about mite allergen exposure and asthma. To develop objective assessments of allergen control procedures, methods and devices. To understand the aerodynamics and distribution of mite allergens. •To facilitate clinical research on the cellular basis of the immune response to dust mites, including T-cell responses, antigen presentation and local immune responses in the respiratory epithelium. To expand knowledge of mite allergen interactions with the innate immune system. •To improve the formulation, reproducibility and potency of mite allergen immunotherapeutics and to develop new strategies for immunotherapy and true prophylactic vaccines.	

## Routes of sensitization and pathogenesis

Sensitization to house dust mites (HDM) in early life is associated with subsequent persistent allergic asthma in childhood and reduced lung function [29, 30]. Although the link between sensitization and allergic disorders is still poorly understood, however, an understanding the nature and mechanism of allergen sensitization provides insights into primary preventive strategies for dust mite allergy.

## Sensitization through the respiratory airway

Being an inhalant allergen, conventional wisdom indicates that the airway mucosa is the main route of HDM allergen sensitization. There is evidence of a dose–response relationship between exposure and sensitization to HDM allergens. However, this dose response relationship is a non-linear bell-shaped curve, with higher concentrations being protective [31]. It has been postulated that the protective effect of high levels of dust mite exposure is related to the concomitant increased levels of immune modifiers, such as endotoxins and fungal beta-glucans [32]. One study showed that the highest rates of sensitization occurred between levels of 3.5 and 23.4 μg/g dust [33]. Hence, it has been suggested that a ‘safe’ level for dust mite avoidance in primary prevention of sensitization studies is a maximum of 2 μg of allergen per g of dust.

HDM sensitization is likely to occur when allergens are airborne, however, measurement of airborne allergens, has been challenging. Although not ideal, the measurement of dust mite allergens in house dust is used as the index of exposure to these allergens. Unlike cat allergen or pollen allergens, dust mite particles are predominantly large particles (>20 μM), and therefore settle rapidly. For example, airborne Group 1 and Group 2 allergens were measurable for only 20 min after agitation or disturbance (eg. cleaning) of dust mite reservoirs [34].

The mechanism by which large dust mite allergen particles reach the respiratory tract to induce sensitization and allergic reactions has been an issue of debate. Nonetheless, it has been demonstrated that minute quantities of dust mite allergen particles that are within the respirable range (1.1 to 4.7 μM) are airborne after disturbance of dust mite reservoirs (eg. by vacuum cleaning without a filter) [35]. The quantity of airborne allergen was however very small and an amplified ELISA system was required to detect these concentrations. This is, however, the likely mechanism by which dust mite allergens reach the lower respiratory tract.

Dust mite allergens are contained in mite fecal pellets and mite body parts. These allergens together with non-allergenic components are powerful inducers of TH2 responses resulting in the induction of IgE antibodies. The list of allergens with inherent adjuvant effects giving rise to IgE sensitization are summarized in Table 3. The immunostimulating effects of these particles arise from the allergens themselves. The major Group 1 allergens (eg. Der p 1 and Der f 1) are cysteine proteases that increase the permeability of the respiratory epithelium by enzymatic digestion of the tight junctions [36]. A similar phenomenon was observed in the skin, where the Der p 1-like cystein protease papain percutaneously led to instant innate inflammation, while notably, specific sensitization was independent on the enzymatic function [37]. More recently Group 2 allergens (eg. Der p 2 and Der f 2) have been shown to be a homolog of the adapter protein MD-2 (a co-receptor of the toll-like receptor) (TLR) that can facilitate lipopolysaccharide-mediated signaling through TLR-4 [18]. Furthermore, these dust mite particles also contain pathogen-associated molecular patterns (PAMPS) such as mite DNA, bacterial DNA and endotoxin, which act to activate the innate immune system and are therefore adjuvants of the allergic response.

**Table 3:** Key dust mite allergen groups with known biochemical identity conferring allergenicity

Allergens Groups	Biochemical Identity	Mechanism of TH2immune induction
Group 1	Cystein protease	Increased permeability through disruption of airway and cutaneous epithelial tight junctions
Group 2 Der p 2, Der f 2	MD-2 like lipid-binding protein	Molecular mimicry of MD2 and DC activation via TLR4 TLR2 activation on DC
Group 3 Der p 3, Der f 3	Trypsin-like serine protease	Increased permeability through disruption of airway and cutaneous epithelial tight junctions PAR-2 activation in airway epithelial cells and keratinocytes
Group 5 Blo t 5, Der p 5, Der f 5	Lipid binding protein	TLR activation?
Group 6 Der p 6, Der f 6	Chymotrypsin-like serine protease	Increased permeability through disruption of airway and cutaneous epithelial tight junctions PAR-2 activation in keratinocytes
Group 7 Der p 7, Der f 7	Lipid binding Protein	Molecular mimicry of lipid binding protein and dendritic cell activation via TLR2, 3, 4
Group 9 Der p 9, Der f 9	Collagenolytic protease	Increased permeability through disruption of airway and cutaneous epithelial tight junctions PAR-2 activation in airway epithelial calls and keratinocytes.

**Fig. 3:**
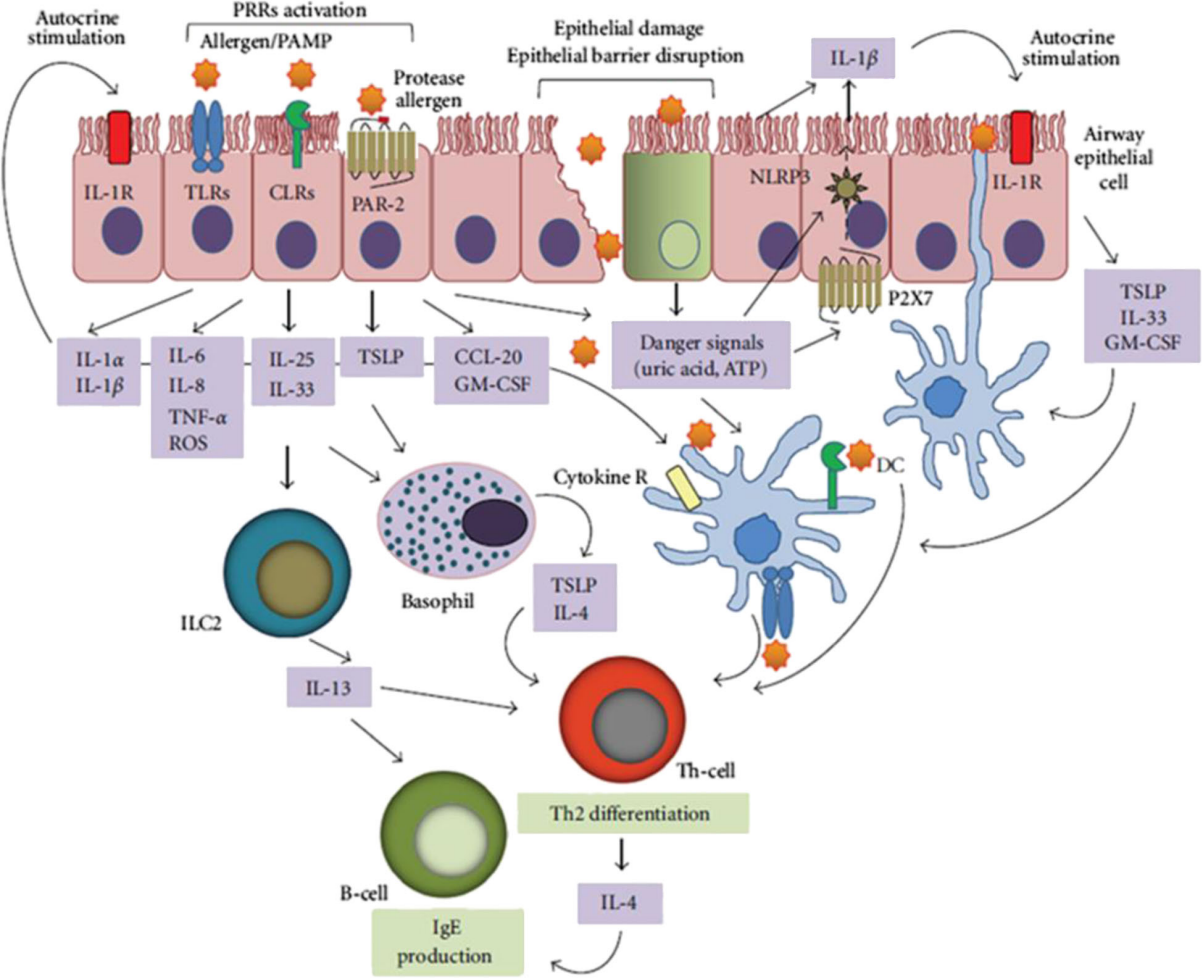
Simplified model of the HDM-induced innate immune activation leading to IgE sensitization in the airways. The induction ofTh2 immunity by HDM allergens results from the stimulation of different innate immune pathways. HDM protease allergens specifically cleave protease sensitive receptors including protease-activated receptor (PAR)-2, disrupt epithelial barrier to gain access to dendritic cells (DCs), and cause tissue injuries to release danger-associated molecular patterns (DAMPs) such as adenosine triphosphate (ATP) and uric acid. Contaminating microbial pathogen-associated molecular patterns (PAMPs) associated or not with lipid-binding allergens trigger numerous pathogen recognition receptors (PRRs) which can produce also DAMPs whereas HDM glycan activation of DCs is mediated through C-type lectin receptors (CLR) ligation. These signaling pathways result in the upregulation of innate cytokines/chemokines such as IL-1*β*, IL-6, thymocyte stromal lymphpoetin (TSLP), IL-25, IL-33, GM-CSF, or CCL20 to recruit and activate inflammatory cells and to induce Th2 differentiation. TSLP mediates OX40L and IL-4 expression in DCs and basophils, respectively, to initiate a Th2-polarized response. IL-25 and IL-33 are strong activators of innate lymphocyte cells (ILC2s) which secrete theTh2 cytokine IL-13 to induce IgE secretion by B cells [40]. Modified from: Alain Jacquet, Innate immune responses in house dust mite allergy. *IRSN Allergy* 2013; 2013: 735031. DOI: 10.1155/2013/735031. The authors used Fig. 2 under the Creative Commons Attribution License

These effects of dust mite allergens on epithelial cells result in the release of epithelial-derived Th-2 promoting cytokines including thymic stromal lymphopoetin (TSLP), IL-25 and IL-33 [38]. A simplified model of HDM-induced innate immune activation leading to dust mite allergen sensitization is depicted in Fig. 3.

## Sensitization through the skin

More recently, the skin has also been recognized as a route of allergen sensitization, particularly when the skin barrier is disrupted by eczema. In support of this, it has been shown that there is a positive correlation between the rate of aeroallergen sensitization and transepidermal water loss (TEWL) in infants with eczema [39]. In fact, filaggrin mutations, which confer susceptibility to eczema, have also been shown to be a risk factor for allergen sensitization [40]. In animal models, overproduction of the cytokine TSLP by eczematous skin promotes airway sensitization to house dust mites, thereby triggering allergic asthma [41]. Taken together, these observations would explain the progression from eczema to asthma, which is known as the atopic march.

The immune mechanisms by which HDM allergens induce a Th2 activity through the cutaneous epithelium are likely similar to those occurring at the respiratory airway. HDM allergens are likely to penetrate the skin barrier via direct proteolytic activity of HDM allergens (eg. Der p 1) and its capacity to bind to lipids (eg. Der p 2). Additional adjuvant properties of allergens and molecules within dust mite particles engage the innate system, particularly through dendritic cells, which results in Th2 skewing and IgE production [42] (Fig. 3).

## Summary

The molecular properties of house dust mite allergens together with exogenous agents contained in dust mite fecal particles render HDM as the source of highly potent allergens. Sensitization occurs mainly through the respiratory tract. However, recent evidence indicates that the eczematous skin is also an important route and may be a mechanism to explain the atopic march.

## Genetics of IgE responses to mite allergens

The specific IgE response is a complex trait highly influenced by the environment. Because of its close relationship with allergic diseases, the genes influencing this phenotype are of great basic and clinical interest. The IgE molecule probably evolved from amphibians IgY [43] and a functional IgE targeting environmental components (allergens) started when an appropriate set of genes controlling specific IgE production was available. Since then, the number of genes influencing IgE has evolved to the current repertoire, which includes some related to general mechanisms of innate and adaptive immune responses and others determining specificity.

IgE hyperresponsiveness to mite allergens is one of the most important asthma-associated phenotypes and is a risk factor for asthma and other allergic diseases. The first attempts to identify genes controlling IgE responses were focused on the Human Major Histocompatibility Complex (MHC/HLA) because of its relevant relationship with the adaptive immune response. The associations of DRB1, DQB1 and DPB1 alleles with almost all kind of allergen extracts and purified molecules have been huge. In 1990, an affected sib pair analysis study showed that IgE hyperresponsiveness to *D. farinae* in patients with allergic asthma was linked to MHC [44]. Several years later, a genome wide search found strong evidence of linkage between the specific IgE responsiveness to *D. pteronyssinus* and chromosomes 6p21 (HLA-D region), 2q21-q23, 8p23-p21, 13q32-q34 and 5q23-q33 in Caucasians families [45]. The role of HLA on the IgE response to this mite was further analyzed by the same authors, evaluating the IgE responses to several allergen components of *D. pteronyssinus* extract [46]. Linkage studies involving HLA loci and mite IgE responses have been replicated in other populations [47, 48].

In a case–control study the frequency of allele HLA-DPB1*0401 was remarkably decreased in patients with IgE hyperresponsiveness to mite allergens, suggesting that it could be suppressing this phenotype in the non-allergic population [49]. In addition, the protective role of other DPB1 allele (*0201) in controlling IgE response to mite has been reported [50]. Other associations include IgE hyperresponsiveness to *B. tropicalis* and *D. pteronyssinus* purified allergens with HLA-DRB1*03 in non-related subjects [51] and family studies [52], although the role of other HLA alleles has also been documented [53–56]. It is worth to mention that the 6p21 region contains additional genes (e.g. butyrophilin-like 2, BTNL2) that have been associated with the risk of mite sensitization [57] but it is unclear whether those associations were due to the linkage disequilibrium with HLA alleles or other yet unclear mechanisms. In populations perennially exposed to mite allergens it is common to find subjects monosensitized to particular mite allergens while others might recognize several allergenic components. The detailed mechanisms explaining how the MHC alleles influence those phenotypes remain to be elucidated. The availability of purified allergen molecules with known clinically relevant epitopes, as well as sequencing approaches for defining HLA alleles, will help to better define the role of this genetic region on the specificity of IgE responses.

As soon as the complex nature of IgE synthesis became more evident, the discovering of “out-MHC” genes influencing IgE was more frequent. Polymorphisms in Th2-genes, for instance those in the gene encoding interleukin 4 at the 5q31 locus [58, 59] and the signal transducer and activator of transcription 6 (STAT6) [60, 61] have been replicated in different populations. Associations with mite sensitization have been also reported with polymorphisms in the genes encoding interleukin-18 (IL18) [62, 63], leukotriene C4 synthase (LTC4S) [64], nitric oxide synthase 1 (NOS1) [65], interleukin 4 receptor alpha (ILR4A) [58], dendritic cell associated nuclear protein 1 (DCNP1) [66], interferon regulatory factor 1 (IRF-1) [67], CD14 [68, 69], Janus kinase 2 (JAK2), GATA binding protein 3 (GATA3), CD40 and interleukin 5 receptor alpha (IL5RA) [70], all of them participating in any of the multiple steps of IgE synthesis. The significant associations with polymorphisms in innate immune genes suggest that genetic effects exert their influences at very early phases of the response. These loci include the complement component 3 (C3) associated with the specific IgE levels to *D. pteronyssinus* [71]; the myeloid differentiation factor 2 (MD-2) associated with the specific IgE levels to Der p 2 [72]; and the nucleotide-binding oligomerization domain containing 1 (NOD1) associated with mite sensitization [70].

For a long time the search for IgE modulating genes has been based mainly on candidate gene approaches, but in the last decade, genome wide association studies (GWAS) and gene expression analyses revealed associations with mite sensitization in new chromosomal regions [73–76] and confirmed the role of previously described HLA alleles [61, 77]. The associations detected by GWAS include the protein kinase domain containing, cytoplasmic (PKDCC) with allergen sensitization in Europeans [73]; thymic stromal lymphopoietin (TSLP) and leucine rich repeat containing 32 (LRRC32) with sensitization to *D. pteronyssinus* and *B. tropicalis* in Singapore (ethnic Chinese) [75]. There are still regions to be fine-mapped because the underlying genes in the associated loci are unknown. That is the case of rs10142119 associated with the sensitization to *D. farinae* in Koreans [76] and rs10174949 associated with mite sensitization in Lithuanians (2p25.1) [78].

Some GWAS pooled individuals with mite-sensitization and those with other specificities in order to increase power; however this makes very difficult to dissect which genes are specifically related with the susceptibility to mite sensitization [61, 74]. GWAS, together with comparative mRNA expression analyses between mite-sensitized asthmatics versus mite-sensitized subjects without asthma, are also revealing divergent gene sets and pathways for these phenotypes [79] in agreement with the fact that sensitization to allergens (atopy) does not necessarily induces allergic symptoms. Nowadays the search for genes controlling the specificity and intensity of specific IgE responses continues, with whole-genome sequencing approaches and the investigation on epigenetic influences on the forefront.

Since allergen exposure varies according to the geographic region, it can be anticipated that genetic epidemiology studies on the same genes but in distinct locations can obtain different results. For example, IL-4 is an important candidate gene for asthma and atopy susceptibility. In Caucasians the effect of IL-4 C-590 T on mite sensitization was dependent of Der p 1 levels. The rare allele T confers a high risk of sensitization only in children exposed to high levels of Der p 1 while the reference allele C was not associated with mite sensitization, independent of the level of allergen exposure [80]. Similar findings have been obtained with the polymorphisms in the gene encoding interleukin 10 (IL-10), which were significantly associated with specific IgE levels to Der p 1 only if effect modification by allergen exposure levels was considered in the model [81].

In addition to the detection of specific IgE to mites as an outcome, the genetic influences on the immune response to mites have been supported by cell assays showing that upon stimulation with mite allergens, the peripheral blood mononuclear cells produce different cytokine levels depending on the carrier status of risk genotypes [82, 83]. Besides, recent studies revealed that epigenetic changes might influence the susceptibility to mite sensitization by modifying DNA methylation in B cells [84], and the hypomethylation of the interleukin 13 gene [85]. Allergen specific immunotherapy has been also found able to change DNA methylation levels at the forkhead box P3 gene (FOXP3) and, by improving the function of T regulatory cells, modify the IgE response to mites [86]. Hence, environmental exposures affecting the epigenome or polymorphisms affecting the interaction between the genome and the epigenetic machinery may play a role in modulating the gene-environment signals that lead to mite sensitization.

Defining the genetic variants underlying complex traits has its intrinsic scientific relevance; in addition, when associated with diseases, polymorphisms are expected to be useful for evaluating the relative importance of the genetic component in multifactorial diseases such as asthma. However, although mite sensitization is one of the most confirmed risk factors for asthma, the impact of the involved polymorphisms in relation to other heritable traits also influencing the pathogenesis of the disease remains to be established, which makes difficult detecting the real effect and heritability of the whole genetic component. Therefore, the usefulness of the variants described in this section as early predictors of mite-induced asthma may be very limited. However, the knowledge of so many variants potentially influencing the IgE response to mite allergens could help to answer fundamental questions of immunology such as the origin of allergenicity.

## Epidemiology

House dust mite sensitization has frequently been shown to be strongly associated with the presentation of allergic airway diseases [87], but its significance varies geographically [88], by ethnicity [89], age-group [90] and environment [91]. Calderon et al. [92] noted that the prevalence of house dust mite sensitization reported in the literature needs to be distinguished between those estimated from unselected (random individuals within) populations and studies focused within groups of selected symptomatic individuals with diagnosed allergic conditions. Additionally, differences in diagnostic tools, end-points, and terminologies used confound the estimates further.

The European Community Respiratory Health Survey I evaluated more than 15,000 adults aged 20–44 years, living in 35 centers in 15 developed countries. This study reported that the prevalence of house dust mite (*Dermatophagoides pteronyssinus*) sensitization assessed via skin prick testing ranged from 4.8% (in Albacete, Spain) to 36.8% (in Hawkes Bay, New Zealand), with a median between centers at 21.7% [93]. In contrast, The First International Workshop on Dust Mite Allergens and Asthma held in 1987 had already reported sensitization figures amongst asthmatics between 45 and 85% while in controls those were 5 to 30% across multiple studies [94]. Amongst the developing as well as tropical regions, Caraballo et al. [95] summarized the sensitization range to be between 10.8% (in a cross sectional population in Butajira, southern Ethiopia) [96] to more than 70% (in Singapore) [97]. While sensitization is highly prevalent in selected populations, only a proportion of them will present with clinical symptoms. Amongst asthmatic cohorts in the tropics, however, it is common to see mite sensitization prevalences of above 80 or even 90% [98, 99].

A key feature of mite sensitization in the tropics is the larger repertoire of specific mite allergens that the atopic individuals are sensitized to [100], possibly due to the presence of a more diverse repertoire of mites being co-dominantly present in the environment (e.g., the concurrent presence of both *Blomia tropicalis* and Dermatophagoides spp.) [100] as well as host genetic factors (with family history being the strongest predictor of allergic diseases) [101]. This is in contrast to the predominant Group 1 and/or 2 house dust mite specific responses in the temperate regions (with more than 70 and 80% of house dust mite allergic patients having specific IgE to these allergens, respectively) [26]. Nevertheless, Batard et al. reported that between 20 and 47% of 1302 house dust mite allergic American, Canadian, European, and Japanese patients evaluated also have IgEs to allergens from groups 4, 5, 7, 13, 15, 21, and 23, and this would have implications for the design, production and standardization of dust mite allergen immunotherapy extracts [26].

To illustrate the natural history of dust mite sensitization, several cohort and cross sectional studies in Singapore are summarized. In a birth cohort in Singapore, the clinical phenotype (eczema and wheeze) with concomitant allergen sensitization in the first 2 years of life were strong predictors of atopic disorders at 5 years [102]. At 3 years of age, 31.4% of atopic individuals in a cross sectional study were already sensitized to house dust mites [103]. House dust mite sensitization in toddlers (age 2–5 years) predict persistent wheeze in children between 8–14 years old [104]. By 14 years of age, the majority of atopic individuals (more than 90%) were sensitized to house dust mites, many of them to multiple species of dust mites [97]. Interestingly, Kidon et al. observed that children with allergic rhinitis and concomitant atopic dermatitis show a preferential sensitization to the Dermatophagoides mites, while *Blomia tropicalis* sensitization is more prominent in children with pure respiratory allergy [105]. In addition, IgE response to a larger repertoire of specific house dust mite allergens is associated with the presence of multi-organ allergic comorbidities (asthma, with allergic rhinitis and/or atopic dermatitis) among children in the tropical environment [100]. It was also observed that migrants originating from non-tropical countries had initially low sensitization rates for house dust mites when they first arrived in Singapore, but these rates increased as they spend more time and reside in the country [87]. This increase was concomitantly accompanied by an increase in airway allergic diseases [87].

Colloff estimated conservatively that 1–2% of the world’s population (65–130 million people) suffer from allergies to house dust mites [106]. This may probably be under-estimated by several folds. Mimicking the significant increase over the last few decades of inhalant allergen induced airway allergic diseases in the “” “Western” world, dust mite associated allergic conditions are also observed to be rising in the developing regions [107] with increased urbanization and adoption of more “westernized” lifestyle. In many of these regions, house dust mites are already the most common cause of sensitization, the specific IgE levels are generally the highest titres, and sensitization to dust mite allergens is usually the strongest predictor of allergic airway disease [87, 103]. With Asia and the developing world making up more than two thirds of the world’s population, dust mite allergy would likely be an even more significant cause of morbidity afflicting billions of people worldwide in the future.

## Clinical pictures: Asthma, Rhinitis/Rhinosinusitis, Atopic dermatitis, Anaphylaxis

### Asthma

Asthma is a major global health problem contributing greatly to socio-economic burden. WHO estimates that it is the most common non-communicable disease among children and, at least, 235 million people currently suffer from asthma worldwide [108].

According to latest GINA definition, “Asthma is a heterogeneous disease, usually characterized by chronic airway inflammation. It is defined by the history of respiratory symptoms such as wheeze, shortness of breath, chest tightness and cough that vary over time and in intensity, together with variable expiratory airflow limitation” [109].

The main causes of asthma are not completely elucidated, but the genetic predisposition of the patient with an appropriate environmental exposure to inhaled substances and pollutants are important risk factors for asthma. Many environmental exposures have been linked to asthma causation, including allergens, tobacco smoke, chemical irritants, pollution, dietary and physical factors as well as respiratory infections [110, 111].

Recently Dick et al. [110] published a systematic review that reports contradictory data regarding HDM exposure and risk for asthma. For instance, Celedon et al. [112] reported that increased HDM (≥10 μg/g) early life exposures was associated with increased risk for asthma at 7 years old (OR 3.0). Three other studies did not find an association between exposure in infancy and asthma at 3, 6–7 or 8 years of age [110].

The effects of HDM on asthma exacerbations and whether interventions aimed at exposure reduction can significantly improve symptoms are also controversial [113].

One of the most important goals now is to identify major settings of exposure and provide feasible interventions to reduce allergen to levels that improve health outcomes. Dust mites tend to live in humid and warm climates and are around all year long but higher in summer due to humidity [114]. Therefore, interventions should be comprehensive and present through different seasons. One recent study looked at different levels of exposure to dust mite in different locations to identify the major sources and settings of exposure that need to be tackled. The highest average exposure (1117 pg/m3, 95% CI: 289–4314) occurred on public transport and the lowest overnight in bed (45 pg/m3, 95% CI: 17–117), which contributed only 9.8% (95% I: 4.415.1%) of total daily exposure. They concluded that the highest levels of exposure to dust mites during 24 h were present in public transportation and the lowest levels occurred during the night in bed. Their results suggest that proximity to people causes more exposure than beds, which is controversial to the previous studies identifying beds as major sources of exposure [115]. This is important from an interventional standpoint because clearly sites with higher exposure need more attention than those with less levels of allergens.

Many studies have looked at the effects of home-based interventions for allergen exposure reduction on asthma improvement and a landmark study identified that a multi-faceted approach in inner-city children was effective in reducing morbidity, cost-effective, and had lasting benefits [116]. Interventions such as caretaker education, use of allergen impermeable covers for mattresses and vacuum with a high efficiency particulate air filter have been shown to help control asthma in patients with dust mite sensitivity [116]. However, a recent study in New York City looked at home based interventions used for exposure reduction to indoor allergens (including but not only dust mite) and compared it to a control group not receiving the intervention. They found that even though household allergens did decrease, the intervention did not reduce the need for asthma medications in already sensitized patients, while it might reduce novel sensitizations. [113].

Despite multiple intervention trials have been conducted there is still some controversy regarding the effects of home-based exposure reduction of allergens on asthma improvement. However, there is less comprehensive data on interventions outside of homes as an important mode of exposure (i.e. schools, work places, day care centers) and asthma control, likely due to challenges of implementing such interventions at those levels. Considering that children spend a great amount of their time in schools, makes schools an important target of intervention. Also finding a feasible intervention at school level can help a group of children as opposed to home-based interventions that can benefit only one or a few children. There have been studies assessing systematic interventions offering medications for asthma control in schools, which proved to be effective [117]. However, there is a need for studies of feasible interventions aimed at reducing environmental exposures such as dust mite in schools in the future.

Group 1 allergens (e.g., Der p1, Der f1, Eur m1) of the different HDM species form a distinct subfamily of C1 cysteine peptidases that are important in the induction of allergic sensitization and asthma [118]. Two general peptidase-dependent mechanisms have been identified. One is the proteolytic attack that cleaves the epithelial tight junctions causing an epithelial barrier damage that facilitates allergen interaction with immune system [119, 120]. The second is the ability of proteases to activate signal transduction pathways of innate immunity that induce recruitment of effector cells and promote a Th2 biased immune response [120]. Based on the role that the peptidase activity of group 1 HDM allergens plays in asthma, a British group has identified a new possible therapeutic approach using specific inhibitors of the HDM peptidases. These new drugs, known as “allergen delivery inhibitors” (ADIs), might provide an effective inhaled treatment for patients suffering from allergic asthma [121].

### Rhinitis/Rhinosinusitis

HDM allergens are highly prevalent, but only a minority of people exposed to them develops clinical symptoms. The prevalence of sensitization to mites is very high (50–90%) in the respiratory allergic population. HDM induced allergic rhinitis typically evolves perennial with seasonal exacerbations in the spring and fall, which corresponds to an increase of proliferation of mites. The persistent nature is more pronounced in temperate regions while the bi-annual rhythmicity (spring and late summer/spring) or intermittent character is more marked in the Mediterranean regions [122]. After a long period of evolution, these features fade and may even disappear, the allergic condition then moving on its own account.

It is generally believed that rhinitis due to HDM allergy is more characterized by nasal obstruction than pollen allergy. However, several studies report all nasal symptom categories [123–125].

The diagnosis is not easy, especially in polysensitized subjects (50–80% of HDM allergic patients). A structured history alone easily misclassifies the allergic status in many allergic patients resulting in false positive rates for HDM allergy of 75% [126], whereas a doctor diagnosis of allergic rhinitis based on the combination of an accurate history, medical examinations and positive skin tests can be confirmed by a positive nasal challenge to HDM in almost all cases [127]. In the absence of possible nasal provocation test, the diagnosis is likely if all of the following signs are present; the diagnosis is possible if any of these signs is absent:

*symptoms are perennial with seasonal exacerbations (Spring, Fall);
*symptoms improve in altitude (>1500 m);
*it is aggravated by contact with household dust and domestic/indoors activities;
*skin prick test to house dust mites extract is positive.

Asthma and rhinosinusitis are common comorbid diseases, without causal relationship with mite hypersensitivity for the latter. In two recent SLIT trials [128, 129], 29–46% of the HDM allergic rhinitis patients included also had asthma. Allergic rhinoonjunctivitis is less frequent. In an analysis of children included in a retrospective HDM SLIT study, the frequency of nasal symptoms ranged from 89.4% for rhinorrhea to 61.7% for nasal pruritus, whereas the frequencies of ocular pruritus and teary eyes amounted to only 25.7 and 17.9%, respectively [125]. However, allergic rhinoconjunctivitis should not be overlooked. Indeed, in a large survey ocular symptoms among allergic rhinitis patients were triggered by house dust and HDM in 34.8% [130]. The study reported a substantial impact of eye symptoms on daily life. In another large observational, prospective and cross-sectional study conducted in France from 2013 to 2014, 56.5% of HDM allergic patients also suffered from conjunctivitis [131]. The strong correlation between allergic asthma and allergic rhinitis as comorbidities is often interpreted as evidence of an underlying common sensitization mechanism.

### Atopic dermatitis

Atopic dermatitis (AD) or atopic eczema, is a prevalent allergic disease leading to significant psycho-social impairment and clinically relevant morbidity. It affects both children and adults in a relapsing course [132]. AD is characterized by a high degree of clinical heterogeneity. Epidemiological studies have shown that IgE sensitization is not causative, but accompanies early skin signs of eczema.[133, 134]

The role of sensitization to inhalant allergens in atopic dermatitis is uncertain. Total serum IgE levels are elevated but seem to be more elevated in AD patients with filaggrin mutations [135].

Is there a role for house dust mites (HDM) in atopic dermatitis?

1. The rate of IgE–mediated sensitization to foods and inhalant allergens is frequent. Most AD patients have elevated levels of serum IgE antibodies specific to HDM allergens; furthermore biopsy specimens of AD lesional skin have been shown to be infiltrated with T lymphocytes that recognize Der p [136].
2. Group 1 mite allergens may facilitate its entry into the skin by enzymatically breaking down the epidermal barrier [137]. This accelerates inflammation, but does not necessarily result in specific sensitization [37]. Mite allergens can activate keratinocytes and induce them to produce and secrete pro-inflammatory cytokines [137–139]. Allergens may sensitize infants with AD via the skin. The proliferation of lymphocytes stimulated with HDM allergens shows significantly higher responses in AD infants than in controls. Serum levels of HDM-specific IgE are significantly correlated with lymphocyte stimulation index. These results support the hypothesis that both food and indoor allergens concurrently sensitize infants via the skin [139].
3. Respiratory allergic diseases due to inhalant allergens are frequent among AD patients.
4. Mites have been found in skin scrapings from patients with AD. Patients with AD showed a higher prevalence of mites on their skin than did healthy individuals, which could be involved in allergic sensitization and disease exacerbation. Though the number of mites on clothes and bedding could be similar in AD patients and controls [140].
5. HDM avoidance measures may reduce the eczema [141]. Avoidance controlled trials have demonstrated that HDM are involved in the pathogenesis of AD in children [142, 143].
6. Epicutaneous application of HDM (Allergen Patch Test, APT) induces AD in nonlesional skin of 50% of AD patients [144, 145]. Thymic stromal lymphopoietin (TSLP), a cytokine produced by epithelial keratinocytes, plays an important role in the pathogenesis of AD. Landheer et al. investigated TSLP expression in nonlesional skin of AD patients following APT with HDM extract. The induction of TSLP protein expression occurred only in patients with a positive APT result, suggesting a role for TSLP in HDM induction of AD-related eczema [146].

Is specific allergen immunotherapy an effective treatment for people with atopic eczema?

Allergen-specific immunotherapy (AIT) has been used to treat mild/moderate AD aiming to restore the imbalance of the immune response [147–149]. A systematic review of randomized controlled trials (RCTs) of specific AIT that used standardized allergen extracts in patients with AD has found limited evidence that specific immunotherapy might be an effective treatment. Subcutaneous and sublingual trials (10/12 trials with HDM) were considered of low quality for the review. Treatment was not associated with increased risk of local or systemic reactions. Future studies should use high quality allergen formulations and should include participant-reported outcome measures [150]. In conclusion, there is evidence that HDM are involved in the pathogenesis of AD but the definite association between HDM allergy and AD still remains to be firmly established.

### Anaphylaxis

Anaphylaxis is an acute emergency that is potentially fatal and commonly related to an allergic and immunologic trigger requiring immediate effective life-saving treatment [151]. Heavy mite exposure in the environment can induce allergic systemic reactions. More recently, the induction of anaphylaxis through ingestion of mite-contaminated foods has been described [152].

Pancake anaphylaxis, also called oral mite anaphylaxis (OMA), is a relatively new syndrome characterized by severe allergic symptoms occurring immediately after eating foods, especially containing flours, contaminated with mites. These cooked foods contain thermoresistant mite allergens and contaminated wheat flour used to make pancakes is its most common presentation [152]. A variant clinical picture is provoked by physical exercise and is called dust mite ingestion-associated exercise-induced anaphylaxis [153]. OMA is more prevalent in tropical and subtropical areas of the globe where mites grow easily in their warm and humid environments [154]. There are reports in the literature of two fatalities associated with the ingestion of foods contaminated with mites [155, 156]. Mites responsible for OMA include domestic and storage species and can be present in any type of flours. There is an intriguing association of OMA and hypersensitivity to aspirin and nonsteroidal anti-inflammatory drugs (NSAIDS) for which there is no good explanation yet and it is more prevalent in patients with house dust mite allergic rhinitis and/or asthma [157]. The higher the contaminated mite ingestion the greater the risk for anaphylaxis. OMA confirmation requires the microscopic documentation and identification of mites in the suspected flour. Alternatively the immunoassay for demonstration of the presence of mite allergens in the suspected flour can be used. It is imperative to try to prevent the worldwide OMA delineating predisposing genetic factors and determining if mite immunotherapy might be efficacious modifying the clinical course of this important variety of food anaphylaxis [152, 158].

Co-sensitization to cockroaches, some crustaceans (shrimp, crab, lobster), shellfish (clams, mussels), and mollusks (snails) is often described and likely due to the presence of allergens in the tropomyosins family, present in some crustaceans (major allergen of shrimp: Pen 1), insects (some flies, mosquitoes, cockroaches), gastropods and mites (Der f 10) [122].

### Occupational diseases induced by mites

Exposure to storage mites has been recognized as an important cause of occupational asthma [159] and rhinitis [160]. An evidence-based review on causative agents of occupational asthma identified storage mites as one of the etiological agents with moderate evidence level in farming and bakery [159].

The main species that have been implicated in work-related rhinitis and asthma are *Lepidoglyphus destructor, Acarus siro/farris, Tyrophagus putrescentiae, Glycyphagus domesticus and Blomia tjibodas* [161]. Allergy to storage mites is relatively common in subjects who work in environments where hay and grain are handled, stored, or processed, such as agricultural workers, farmers, millers and bakers [162]. In addition, other professions, particularly in the food industry, like poultry and ham workers, can also develop sensitization and respiratory symptoms due to storage mites [163]. IgE-mediated allergy to storage mites has been demonstrated by skin prick testing and measurements of allergen-specific IgE, and confirmed by specific inhalation challenges [164, 165]. The occurrence of nasal symptoms has been found to precede the development of lower airway symptoms [160].

Although work-related allergic reactions to mites have been mainly attributed to mites (storage), there is increasing evidence showing that house dust mites (Dermatophagoides spp.) are potential work-related risk factors [166]. Several studies have reported high levels of domestic mite allergens in occupational settings, particularly in schools and day care centers, but clinically relevant exposures can also been found in various workplaces, including poultry farms, hotels, cinemas, libraries, public transportation, fishing-boats, submarines, and churches [166].

Sander and coworkers have assessed domestic mite allergens in floor and airborne samples from workplaces and living areas using a sensitive immunoassay to measure personal airborne mite allergen exposure [167]. These authors found that inhalable dust mite allergen concentrations in most of the workplaces investigated were higher than those in living areas, and significant differences were found for textile recycling, bed feather filling, feed production, grain storage and cattle stables [167]. These studies [166, 167] show that exposure to house dust mite allergens can be higher in occupational than in domestic settings, and therefore subjects sensitized to house dust mites can experience worsening or aggravation of respiratory symptoms in the workplace, leading to work-exacerbated asthma.

Regarding the importance of domestic animals in mite sensitization, pets (dogs, cats) are important carriers and reservoirs of HDM and interestingly, D. farinae is much more important as an allergen for allergic canines themselves than D. pteronyssinus, whereas the major allergens have been defined as Der f 15 and Der f 18 on the molecular basis [168].

Spider mites (family Tetranychidae, suborder Prostigmata, order Acari) are outdoor phytophagous mites that damage fruit leaves. Several case reports and cross-sectional surveys have demonstrated that spider mites are important causative allergens of rhinitis and asthma in fruit farmers and greenhouse workers [169]. The two-spotted spider red mite (Tetranychus urticae) is the most common in pear farms, greenhouses and herbaceous plants, whereas the European red mite (Panonychus ulmi) is the most frequent pest in apple farms, and the citrus red mite (Panonychus citri) is usually found in citrus farms and orange groves.

Predatory beneficial mites, such as *Amblyseius cucumeris* and *Amblyseius californicus*, are increasingly being used as biological control measures in horticulture, and these mites have been shown to give rise to IgE-mediated sensitization and skin rashes, conjunctivitis, rhinitis [170] and occupational asthma [171] among exposed greenhouse employees.

### Diagnosis and usefulness of component resolved diagnosis (CRD). Molecular diagnosis

Diagnosis of house dust mite (HDM) allergy is routinely performed with crude mite extracts which contain a mixture of allergenic and non-allergenic components in variable amounts and are only standardized for group 1 and/or group 2 allergens. Other important allergens, e.g., Der p 23 [172, 173] are only present in small amounts in many commercial HDM extracts and often not detectable [174]. Therefore, patients without sensitization to group 1 or group 2 allergens are often not diagnosed with mite extracts and diagnosis with mite extracts cannot determine the allergens which are responsible for the allergy [174]. A molecular diagnosis with all important HDM allergens allows the diagnosis of all HDM allergic patients and the determination of the exact sensitization profile of a patient, thus determining the disease-causing allergens. Molecular diagnosis of HDM allergy can be performed by ImmunoCAP (Thermofisher, Uppsala, Sweden) for nDer p 1, rDer p 2 and the mite tropomyosin, rDer p 10, or by allergen microarray chip (ImmunoCAP ISAC Test, Thermofisher), which contains the same HDM allergens. However, not all HDM allergic patients can be diagnosed with these allergens and it has been shown that 5% of HDM allergic patients are monosensitized to Der p 23 [175].

Recently, within the MeDALL (Mechanisms of the Development of ALLergy) project, a customized allergen-chip was developed which contained the most important HDM allergens (Der p 1, 2, 4, 5, 7, 10, 11, 14, 15, 18, 21 and 23) [176, 177] and which was comparable to skin prick testing and ImmunoCAP for diagnosis of allergic rhinitis and asthma [178]. A component-specific diagnosis allows the determination whether a patient is genuinely sensitized to HDMs (e.g., IgE-reactivity to Der p 1 or Der p 2), or if the reaction to HDMs is caused by cross-reactivity (e.g., exclusive sensitization to Der p 10 in shrimp allergic patients) [179]. Additionally, using molecular diagnosis it was possible for the first time to answer the question whether IgE reactivity profiles change substantially or remain constant over time, showing that de novo sensitizations to new allergens are rare events in allergic adults [180]. A long lasting goal of allergy research was to find markers which may predict the development of allergy in children and the development of severe forms of disease manifestations (e.g., asthma, atopic dermatitis). Using component-specific diagnosis, it was shown that allergic rhinitis to birch pollen in adolescence can be predicted by IgE reactivity to pathogenesis-related class 10 proteins in early childhood [181] and that sensitization to Fel d 1 and Can f 1 in childhood were associated with symptoms to cat and dog in adolescence [182]. Likewise, high Ara h 2 IgE titers were shown to be associated with peanut-induced anaphylaxis [183]. In the case of mite allergy, CRD with the MeDALL chip indicated that IgE responses to mites were initiated by Der p 1, Der p 2 or Der p 23 and increased in prevalence along the first decade of life [184]. Additionally, early IgE sensitization to Der p 1 and to Der p 23, but not to Der p 2, was significantly associated with asthma at school age and children with broad IgE sensitizations to mite allergens had a significantly higher risk to develop asthma [184]. In this context, it was also shown that mite allergic children with asthma reacted with more HDM allergens than children without asthma and had also higher IgE-levels to the individual allergens than the children without asthma. In contrast, fewer asthmatic children showed IgG reactivity to HDM allergens than non-asthmatic children. In the case of atopic dermatitis, CRD showed that allergens which were only found in mite bodies (i.e., Der p 10, 11, 14 and Der p 18) were more often recognized by HDM allergic patients with atopic dermatitis than by patients with respiratory symptoms, whereas no difference in the frequency of IgE reactivity was found to allergens derived from faeces (i.e., Der p 1, 2, 5, 7, 21 and Der p 23) between patients with atopic dermatitis and patients with respiratory allergy [185]. Therefore, it is possible that atopic dermatitis patients become sensitized to mite body-associated allergens by skin contact. In a recent study, it was also shown that allergen contact by the oral route induced preferentially IgG responses whereas respiratory allergen contact was important for IgE sensitization [186].

### Specific immunotherapy with mite allergens

Allergen Immunotherapy (AIT) is widely used in clinical practice for patients with house dust mite (HDM) allergy. AIT may be delivered via subcutaneous (SCIT) and sublingual routes (SLIT) [187]. AIT is a therapeutic intervention which is adapted to the specific IgE spectrum of an individual and modifies the natural course of the disease as it has a persistent efficacy after completion of treatment. In this perspective, AIT has to be presently considered a prototype of Precision Medicine [188].

Despite the global use of HDM-AIT, documentation on its efficacy has been controversial [189]. This is mainly due to the clinical and methodological heterogeneity amongst studies [122]. Generalised conclusions (“class effects”) on the efficacy and disease-modifying effects to all AIT products are unjustified. In contrast, each product needs to be evaluated individually, based on available study results, to justify efficacy and specific claims on sustained and disease modifying effects per allergen and targeted patient group (children vs. adults, allergic rhinitis vs. asthma) [190].

### Allergic Rhinitis

**Fig. 4:**
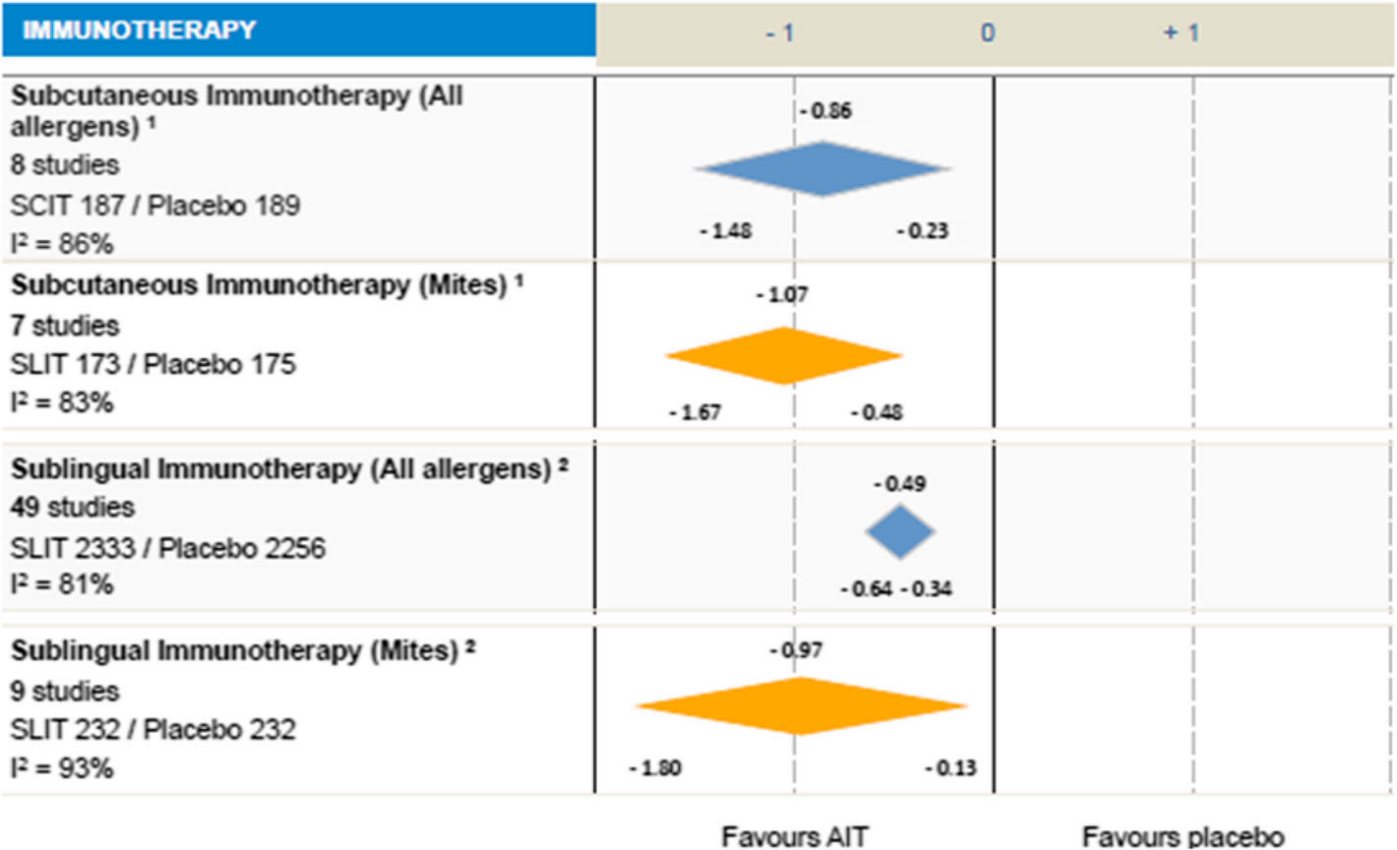
Allergen Immunotherapy for HDM-induced Rhinitis. Adapted from: Moisés A. Calderón, Jörg Kleine-Tebbe, Allan Linneberg, Frédéric De Blay, Dolores Hernandez Fernandez de Rojas, Johann Christian Virchow, Pascal Demoly. House Dust Mite Respiratory Allergy: An Overview of Current Therapeutic Strategies The Journal of Allergy and Clinical Immunology: In Practice 2015; 3(6): 843–855. http://dx.doi.org/10.1016/j.jaip.2015.06.019. Adapted under the Open Access Creative Commons License. The authors have used Fig. 3a, Immunotherapy. Calderon MA et al. J Allergy Clin Immunol In Practice 2015; 3: 843-55. ^1^Calderon MA et al. Cochrane Database of Systematic Reviews 2013. No.: CD007163[*In prees*]. ^2^Radulovic S et al. Cochrane Database Syst Rev. 2010 Dec. 8:(12):CD002893

AIT has been included as a therapeutic intervention in the management of AR in the ARIA guidelines. Evidence on AIT efficacy has been thoroughly evaluated. In the sub-analysis of the SLIT Cochrane systematic review and meta-analysis, Radulovic et al. [191] found a significant reduction in symptom scores (SMD −0.97; CI −1.8 to −0.3; *p* = 0.02; *n* = 464 patients) and in medication scores (SMD −0.52; CI −1.09 to −0.03, *p* = 0.07; *n* = 189 patients) compared with placebo (Fig. 4).

Recently, a pan-European double-blind placebo-controlled randomized controlled (DB PC RCT) trial that included 992 adults with moderate-to-severe HDM-induced AR despite treatment with pharmacotherapy was published [192]. Analysis of the primary end point demonstrated absolute reductions in total combined rhinitis score of 1.18 (*p* = 0.002) and 1.22 (*p* = 0.001) compared with placebo for 6 SQHDM and 12 SQ-HDM, respectively. The statistically significant treatment effect was evident from 14 weeks of treatment onward. The treatment was well tolerated.

The safety of the new SLIT-HDM tablet has been evaluated in a multicentre, DB PC RCT in North American children 12 to 17 years old with HDM AR with and without conjunctivitis and with or without asthma [193]. The 6 and 12 SQ-HDM doses of the HDM SLIT-tablet were well tolerated, and local AEs were of short duration. No anaphylactic reactions, systemic allergic reactions, AEs requiring epinephrine, serious AEs, or local swellings in the mouth or throat assessed as severe were reported.

### AIT in allergic asthma to HDM

Although AIT is widely used in children and adults with HDM-Asthma, the level of evidence for its efficacy and safety is still a matter of debate. Many different individual publications and systematic reviews have shown superiority of SCIT [194] and SLIT [195] over placebo. However, the majority of these studies were heterogeneous for allergen dose, duration, and patient selection.

**Fig. 5:**
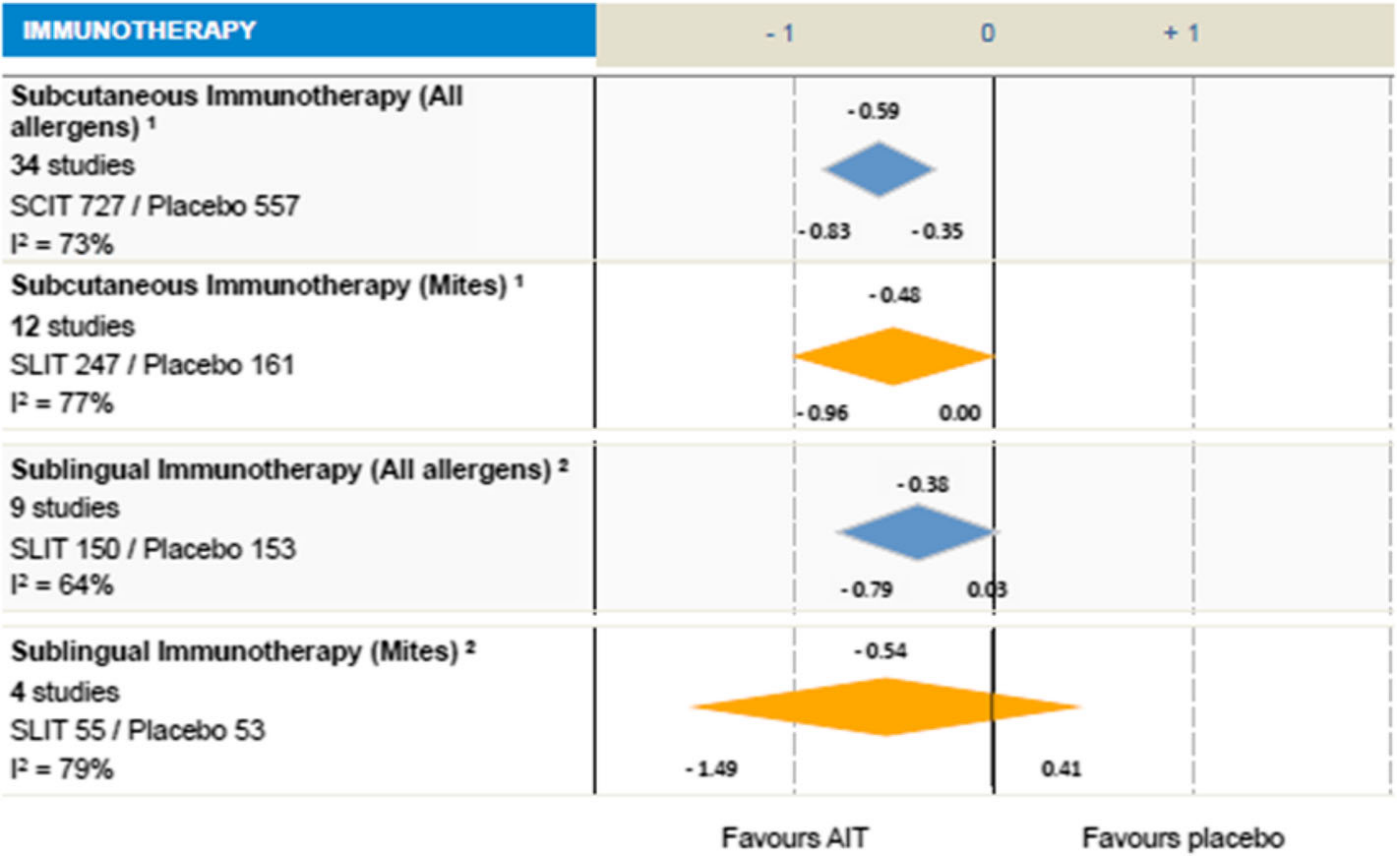
Allergen Immunotherapy for HDM-induced Asthma. Adapted from: Moisés A. Calderón, Jörg Kleine-Tebbe, Allan Linneberg, Frédéric De Blay, Dolores Hernandez Fernandez de Rojas, Johann Christian Virchow, Pascal Demoly. House Dust Mite Respiratory Allergy: An Overview of Current Therapeutic Strategies. The Journal of Allergy and Clinical Immunology: In Practice 2015; 3(6): 843–855. http://dx.doi.org/10.1016/j.jaip.2015.06.019. Adapted under the Open Access Creative Commons License. The `s have used Fig. 3b, Immunotherapy. Calderon MA et al. J Allergy Clin Immunol In Practice 2015; 3: 843-55. ^1^Abramson MJ et al. Cochrane Database Syts Rev. 2010 Aug 4: (8): CD001186. ^2^Calamita Z et al. Allergy 2008 ; 62: 1162-72

For SCIT, in a sub-analysis of 12 studies [194] a reduction in asthma symptom scores (SMD −0.48; CI 95% –0.96 to −0.00; n = 408) and asthma medication scores (SMD −0.61; CI 95% –1.04 to −0.18; n = 424) was found. For SLIT, nine DB PC RCT studies evaluated the effect of HDM-SLIT over placebo [195]. The authors found a significant reduction in asthma symptoms (SMD −0.95; CI 95% –1.74 to −0.15, *p* = 0.02; *n* = 243 patients) and a reduction in rescue medication use (SMD −1.48; CI 95% –2.70 to −0.26; *p* = 0.02; *n* = 202 patients) (Fig. 5).

The efficacy and safety of the new HDM-SLIT tablet was first evaluated as steroid sparing treatment while keeping asthma control [196]. More recently, in an European double-blind placebo-controlled randomized trial 834 adult patients with HDM-AA and HDM-AR, which were not well controlled by inhaled corticosteroids (ICS) were included [197]. Patients were treated daily for 12 months with either a 12 SQ-HDM or a 6 SQ-HDM dose, or with placebo in addition to ICS and short-acting beta-agonists (SABA). The trial showed that 12 SQ-HDM (the dose approved in the EU) significantly reduced the risk of a moderate or severe asthma exacerbation relative to placebo with a hazard ratio (HR) of 0.66, corresponding to a 34% risk reduction. This includes a 36% reduction in risk of nocturnal awakening or increase in daily symptoms (HR: 0.64) and a 48% reduction in the risk of increased use of SABA treatments (HR: 0.52) [197].

### AIT in atopic dermatitis to HDM

At present, few studies have evaluated the efficacy of AIT (both SCIT and SLIT) in atopic dermatitis (AD). Some studies have demonstrated good efficacy effect, although there are conflicting data on which patient population benefits, for example, moderate versus severe AD. The clinical and methodological heterogeneity in these studies is a limitation to make comparisons, thus, nearly impossible to base conclusions about the treatment efficacy. More research is needed with randomised trials of well-defined patient populations with AD using uniform, standardized outcomes to establish comparative effectiveness of AIT in AD [198].

### Prevention of Dust Mite and Dust Mite Allergen Exposure

#### Justification for Dust Mite Exposure Control

The decision to initiate environmental controls to reduce dust mite exposure can be complex. Total prevention of exposure to mite allergenic material to prevent IgE sensitization to mite allergens in genetically susceptible individuals requires strict, continuous avoidance of mite exposure, which is practically all but impossible [199]. Furthermore, to curtail development of all cross-reacting specific IgE, avoidance of all arthropods would probably be required [200]. The majority of the world’s population lives on seacoasts [201] or along rivers [202] and these areas typically have adequate humidity to support growth of dust mites and storage mites during all parts of the year.

Much research has been conducted to determine if it is possible to reduce development of mite-specific IgE-mediated sensitization (primary prevention). Several studies comparing dust mite sensitization rates in children from areas endemically low and areas endemically high in dust mite allergen indicated that the prevalence and degree of sensitization to dust mite was strongly associated with the amount of exposure to mite allergens [203, 204]. A prospective study of mite allergen avoidance in Manchester, UK, [205, 206] using a combination of interventions, decreased Der p 1 from mattresses by 97% to the nanogram range during pregnancy and 12 months after birth in the active group [205]. However, with all possible dust mite exposures at homes of friends and family, on public transportation and in public places and at schools and day care centers, primary prevention of dust mite sensitization by mite allergen avoidance may not be possible [207–209].

Secondary prevention, or the attempt to reduce the risk of asthma in dust mite sensitized children has also received much attention. The link between asthma and dust mite exposure is one of the most extensively studied relationships between environmental exposure and disease development [210–213]. In all climates conducive to the growth of dust mites, mite exposure may be one of the factors contributing to the development of asthma [112, 214]. Secondary prevention has also been the goal for many children with allergic rhinitis who are at risk of the subsequent development of asthma. However, to date there is no evidence-based information as to whether mite avoidance may be effective as a secondary preventive measure to prevent/delay asthma development among mite-sensitized individuals, or those with allergic rhinitis.

The relation of dust mite allergen exposure and the worsening of allergic respiratory symptoms is well documented [215]. In one study of 311 subjects both sensitized and exposed to high levels of indoor allergen including dust mite allergen there was significantly lower FEV_1_% predicted values (mean, 83.7% vs 89.3%; mean difference, 5.6%; 95% CI, 0.6%-10.6%; P = .03), higher eNO values (geometric mean [GM], 12.8 vs 8.7 ppb; GM ratio, 0.7; 95% CI, 0.5-0.8; P = .001), and more severe airways reactivity (PD_20_ GM, 0.25 vs 0.73 mg; GM ratio, 2.9; 95% CI, 1.6-5.0; P < .001) as compared with subjects not sensitized and exposed [216]. Adults in a 4-year study who were both sensitized and exposed to high levels of dust mite allergens had increased bronchial hyper-responsiveness [217]. Many additional links between dust mite exposure and allergic disease are documented in the recent environmental practice parameter on dust mites [198]. A reduction in the symptoms experienced by those with atopic dermatitis has also been linked to house dust-mite allergen avoidance [218].

#### Facilitative factors and Allergen Reservoirs

Controlling factors that facilitate the growth and reproduction of dust mites has been an often sought goal in exposure control. The dependence of dust mites on the water content of the air has been extensively documented [219, 220]. Arid climates have an intrinsically low abundance of dust mites, and the most effective method of controlling dust mite exposure is to live in a very dry climate such as the high desert of New Mexico in the US or the Altiplano or Bolivian Plateau, in west-central South America [202]. Since this is not a practical solution, mimicking these conditions in the home environment as much as possible provides an opportunity to control mite population growth.

Humidity control should be the mainstay of any mite control efforts. The most important factor facilitating dust mite growth, reproduction and allergen production is the availability of water in the surrounding environment [220]. Mites absorb moisture directly from their surroundings under conditions of high moisture and lose water when moisture is low. The mite moisture equilibrium therefore is not directly relative humidity dependent. It is instead dependent of the moisture situation of the local microenvironment and the moisture retention ability of the mite’s immediate surroundings such as carpet dust reservoirs or bedding. A simple measurement of relative humidity may not assure an environment free of dust mite activity. Microenvironments that exist in bedding, in carpet next to concrete or in pet lounging areas may provide adequate moisture for mite survival in climates not expected to have a mite presence. A mite surrounded by a hygroscopic microenvironment as moist bedding can survive much dryer conditions than would be expected. Of note, exposure to a moisture rich environment for only a short period can provide enough moisture for growth and metabolism [221].

Although directly linked to water content of the air in the calculation of relative humidity, temperature is also a factor in dust mite survival. Conditions at the extreme ends of the temperature spectrum, either to cold or to hot can impact mite survival although elevated temperature conditions tend to be more lethal than freezing. Mites and their eggs survive poorly when exposed to hot water and clothes dryers but survive during short periods of freezing conditions. The exposure to direct sunlight is an often forgotten factor in the destruction of dust mites [222].

It is not enough to address mite factors facilitating mite population growth. Reservoirs of mite allergen must also be eliminated. House dust mites can be found in any area of the home, however they are most often associated with certain indoor environments including the bedroom carpet, mattresses and bedding, frequently occupied upholstered furniture and in pet lounging areas [223, 224]. Recent investigations have questioned the traditional concepts of the location of dust mite reservoirs indicating that significant exposure can occur in public transportation conveyances and associated with work environments as well as clothing [207].

#### Climate Factors

Although residents of cold and arid climates are less likely to be exposed to house dust mites, the large majority of the world population is exposed to house dust mites. Nearly half of the people in the world live within 200 km of the coast where humidity levels are typically higher. The rate of population growth in coastal areas is accelerating. In China alone over 400 million live in coastal cities. Dust mite exposures and the allergic problems related to those exposures are likely to increase [201].

Although many climates are naturally conducive to mite growth and allergen production, the artificial control of indoor climates is increasing. Even though it is energy intensive, the use of forced air heating and air conditioning is growing around the world and especially in more affluent economies. Dust mite allergen exposure control is therefore a viable option for large numbers of persons. In many areas seasonal heating requirements result in very dry indoor environments and subsequently dust mite exposure is a seasonal phenomenon. Low humidity conditions can also be obtained through use of air conditioning and dehumidification. Yet, in many areas of the world ambient humidity levels are high enough that producing low humidity levels sufficient to preclude dust mite growth is not practically achievable. The recent Cochrane study on dehumidification alone indicates that evidence of clinical benefits of dehumidification using mechanical ventilation with dehumidifiers is scanty [225]. Indeed, the meta-analysis of multiple dust mite control studies would lead the reader to believe that there is nothing that can be physically done to control dust mites and improve health. Yet, this conclusion is disputed by many experts in the field of allergy [226]. Furthermore, the nature of single source exposure control studies may preclude successful clinical improvement because allergen sensitization is typically to multiple agents.

A significant amount of work has been done on removal of mites and mite allergens through cleaning. It goes without saying that efforts to control mite infestations of the skin and remove mite infestations from clothing are essential in the maintenance of overall health [227]. Humans have been living with dust mites for generations and they might even be described as among our “old friends” [228]. But no physician would advocate for wearing mite infested clothing or sleeping in mite infested bedding. Mite sensitization is likely to occur in genetically susceptible individuals, therefore efforts to reduce instances of elevated mite exposure and thus reduce allergic symptoms are only prudent [229].

Since mite allergens are located in known areas of a typical house [229, 230] removing mite allergen reservoirs is a very effective way to reduce mite allergen exposure. Efforts to remove carpets, drapes, upholstered furniture and any other fabric covered objects from the living environment can effectively reduce mite allergen exposure. The extent to which these items are removed will ultimately be a matter of personal preference. Since mite allergens are known to be heavy and not aerodynamically suited for airborne disbursal [34] and high humidity microenvironments are known to exist in bedding it is logical to focus dust mite reduction efforts on bedding. Efforts to enclose mattresses, box springs and pillows in mite-impermeable covers are known to be very effective [231]. However, it is important to mention that the efficacy of allergen avoidance in patients with already established rhinitis or asthma is a matter of debate [232–235].

Washing bedding in hot water and even with bleach and drying bedding in very hot conditions or even in direct sunlight are known to reduce both the presence of mite allergen and the mites themselves [236, 237]. Washing bedding and clothing removes mite allergens and kills mites. Most of the killing is through drowning, although washing in hotter water kills more mites. The temperature used to wash bedding has become an issue. Elevated temperatures are more energy intensive and hotter water is a scalding hazard. Experts agree that washing is better than not washing and washing with water that is 48° Celsius provides optimum mite killing and home safety [199].

Heat treatment can be effective in killing mites and their eggs. Treatment of cloth at 95° Celsius killed all mites present [238]. However, treatment at 40 °C under dry and wet conditions allowed approximately 80% of all mite eggs to survive. Under dry heat at 50 °C, the thermal death point of dust mite eggs occurred at 5 h and at 60 °C death occurred almost instantaneously [239]. Presumably the eggs survive heat better than the mites themselves. Homes treated with heat-steam over a period of months showed a sustained reduction of Der p 1 and Der p 2 compared to sham treated homes [240] However, mite allergens have been demonstrated to be stable even at elevated temperatures [241].

Although the practice has fallen into senescence in the modern world of appliances, there was a time when frequently placing bedding in direct sunlight for several hours was practiced in many cultures. It has been demonstrated that ultraviolet irradiation is lethal to many organisms including dust mites [242, 243].

Many harsh chemicals are known to kill dust mites or denature mite allergens in industrial and household settings. Agents like tannic acid, Benzyl benzoate, Disodium octaborate tetrahydrate, tri-n-butyl tin maleate, pirimiphos methyl and even “essential oils” like methyl eugenol have been described in the literature to effectively kill mites [244–248]. However, they are all dangerous at some concentration and cannot be recommended for use by patients or homeowners [199].

It has been suggested that freezing can be effective in killing dust mites and the recommendation to place small cloth items like stuffed animals in the freezer compartment of house hold refrigerators has been frequently given out by allergists. However, there is little evidence that this is effective. There may be some mite death due to desiccation in the dry environment of a household freezer. But, dust mite eggs have been shown to resist freezing at temperatures above −70° Celsius [222]. And, freezing is not effective in removing dust mite allergen from reservoirs because dust mite allergen is stable at low temperatures for extended periods of time [239].

Air conditioning would have a twofold impact on dust mite populations. The cool temperatures will slow mite metabolism and reproduction and reduce moisture need for mite survival. Microenvironments or increased humidity can be reduced using a dehumidifier and/or air conditioning. The absence of air conditioning has been shown to be a factor contributing to increased mite allergen levels in US homes [249]. Air conditioners must be operated for a long time to remove sufficient moisture from the air to effectively decrease room humidity. Mechanical ventilation heat pump recovery units in the UK failed to achieve the desired mite reduction results [250].

Evidence on clinical benefits of dehumidification using mechanical ventilation with dehumidifiers remains scanty [225]. Although dehumidification and air conditioning doubtlessly reduce overall dust mite exposure [251], the difficulty in using dehumidification alone in damp environments to decrease dust mite antigen exposure has been described in a recent Cochrane review [225].

## Summary of current recommendations

Most publications on allergy and dust mite control would agree that a comprehensive program of personal hygiene, bed hygiene, properly fitted allergen-impermeable covers, cleaning, dehumidification or air conditioning and appropriate food storage in very damp climates can reduce exposure to house dust mite allergens. It is a stretch further to conclude that the above steps can improve symptoms in those already allergic to dust mites. However, depending on the sensitivity and life style of the allergic person, prudent efforts over an extended period of time are likely to result in gradual improvement in health. The fact that current studies do not provide sufficient evidence for critical reviews to conclude there is unequivocal benefit is no reason to abandon logical and prudent efforts to reduce mite exposure.

### Unmet Needs in Mite Allergy Research

The authors view the following as currently unmet needs in mite allergy research:

- Since mites constitute the most important allergen source worldwide the information contained in this document needs to be disseminated to all ranks of the medical establishment for educational purposes and to stimulate research
- Increased knowledge on the cellular basis of the immune responses to mites
- A better understanding of the link between mite sensitization and allergic diseases
- Better insights into the genetic influences controlling IgE responses to mite allergens. Effects of epigenetic factors
- Improved mite allergen standardization
- Development of purified mite allergens with defined clinically relevant epitopes for molecular diagnosis and evaluation of the response to immunotherapy
- Development of objective methods to assess allergen exposure and environmental control outcomes
- Better strategies for immunotherapy and immunoprophylaxis of mite allergy: recombinant allergens, hypoallergens, T cell peptides.
- Performance of controlled immunotherapy trials with high quality allergen formulations using standardized outcomes
- New strategies for primary and secondary prevention of mite-induced diseases
- Primary prevention efforts might be strengthened with a better understanding of the link between mite sensitization and allergic disease

## Abbreviations

AAAAI: American academy of allergy asthma and immunology
ACAAI: American College of Allergy Asthma and Immunology
DCNP: Dendritic Cell Associate Nuclear Protein
EAACI: European Academy of Allergy and Clinical Immunology
ELISA: Enzyme-linked immunosorbent assay
GWAS: Genome Wide Associations Studies
HDM: House dust mite
iCAALL: International Collaboration in Allergy Asthma and Immunology
ICON: International Consensus on …
IL: Interleukin
IRF: Interferon regulatory factor
IUIS: International union of immunological Societies
JAK: Janus kinase
LPS: Lipopolysaccharide
LTC4S: Leukotriene C4 synthase
MARIA: Multiplex array for indoor allergens
MHC/HLA: Human major histocompatibility complex
NOS: Nitric oxide synthase
PBD: Protein database
PKDCC: Protein kinase domain containing, cytoplasmic
RAST: Radioallergosorbent test
STAT6: Signal transducer and activator of transcription 6
TEWL: Transepidermal water loss (TEWL)
TLR-4: Toll like receptor 4
TSLP: Thymic stromal lymphopoietin
WAO: World Allergy Organization
WHO: World Health Organization
